# Expression of hormonal receptors and Toll-like receptors in cultured canine uterine explants with pseudoplacentational endometrial hyperplasia and bacterial-elicited endometrial inflammation

**DOI:** 10.1371/journal.pone.0331209

**Published:** 2025-09-05

**Authors:** Clarissa Helena Santana, Monique Ferreira Silva Souza, Laice Silva, Lucas dos Reis Souza, Ayisa Rodrigues Oliveira, Álan Maia Borges, Tatiane Paixão, Renato Lima Santos

**Affiliations:** 1 Departamento de Clínica e Cirurgia Veterinária, Escola de Veterinária, Universidade Federal de Minas Gerais, Belo Horizonte, Minas Gerais, Brazil; 2 Departamento de Patologia Geral, Instituto de Ciências Biológicas, Universidade Federal de Minas Gerais, Belo Horizonte, Minas Gerais, Brazil; ICAR-Indian Veterinary Research Institute: Indian Veterinary Research Institute, INDIA

## Abstract

Pseudoplacentational endometrial hyperplasia (PEH) is a common uterine lesion in dogs. A high frequency of pyometra has been associated with PEH in dogs, suggesting that PEH might be related to the pathogenesis of pyometra. This study aimed to assess transcription levels and expression of Toll like receptors (TLR) 1, 2 and 4; alpha estrogen receptors (ESR1), progesterone receptors (PR) and prolactin receptors (PRLR) in uteri with PEH. Furthermore, the inflammatory response of dog endometrium with PEH against *ex vivo* bacterial stimulus was also investigated. Uteri were classified as controls or with PEH. Uterine explants were cultured for 6 and 12 hours after in vitro stimulus with inactivated *Escherichia coli*. Transcription of receptors and proinflammatory cytokines, namely interleukin-6 (*IL-6*) and *CXCL8* were evaluated. Expression of receptors was also evaluated in uterine explants and uteri from biopsy archives. CXCL8 concentration was measured in supernatants from all cultured explants. Transcription levels and expression of both PR and ESR1 were lower in uteri explants with PEH not stimulated and cultured for 6 hours. Expression of PRLR was higher in uteri with PEH from biopsy archives. Proinflammatory response by transcription levels of interleukin 6 demonstrated downregulation in uteri with PEH at 6 hours of stimulation followed by upregulation at 12 hours. However, no differences between groups were observed. Both control and uteri with PEH secreted similar concentrations of CXCL8 at 6 hours of bacterial stimulation. At 12 hours, no response to stimulation was observed in the PEH group and supernatant concentrations of CXCL8 were higher in the control group. The inflammatory response to bacterial stimulus in uteri with PEH had different pattern than the control group, with an inversion in *IL-6* transcription levels between 6–12 hours of culture. Additionally, CXCL8 production ceased earlier in explants with PEH than in control.

## Introduction

Pseudoplacentational endometrial hyperplasia (PEH) is a uterine hyperplasic change that affects female dogs. It is characterized by endometrial hyperplasia with morphologic features that resemble placentation sites [1,2]. PEH was formerly named deciduoma [3] until Schafler and Gifford [1] provided a thorough morphologic description of this change and renamed the condition as PEH. Although very common, not much is known about the pathogenesis of PEH and its consequences. Indeed, this condition is still largely unknown by many clinicians and pathologists [4–8]. PEH occurs during the diestrus, when there are higher plasmatic progesterone concentrations, particularly during the first half of the diestrus. Repeated exposure to high progesterone in successive estrous cycles without breeding predisposes hyperplastic changes in the endometrium [1,7]. Incidence of PEH ranges from 40% to 80%, and predisposing factors for PEH have been recently described [29]. This condition is significantly more frequent in dogs between 4 and 12 years of age, whereas cystic endometrial hyperplasia (CEH) is significantly more frequent in dogs older than 12 years, and the frequency of PEH was significantly higher than that of CEH. Breed predisposition have also been identified with Shih-Tzus having the highest frequency of PEH, but there is no breed predisposition for CEH [29]. Importantly, PEH affects non-pregnant dogs and differs morphologically from CEH. In PEH there are morphologic changes in the superficial endometrial epithelial cells, which have an expanded and finally vacuolated cytoplasm characterizing decidual reaction that mimics maternal placental tissues [1,2], another hyperplasic uterine lesion that also occurs during the diestrus [9]. PEH is unrelated to subinvolution of placental sites, which may occur after parturition [10]. It has been hypothesized that PEH is a lesion associated to the clinical manifestation of pseudopregnancy (also known as pseudocyesis) in dogs [11]. However, this association between PEH and pseudopregnancy has not yet been demonstrated.

Pyometra is an important and potentially life-threatening uterine inflammatory disease for female dogs. It is characterized by intrauterine accumulation of purulent exudate and systemic illness due to endotoxemia [12–14]. Although the pathogenesis of pyometra is still not completely clear, it is known that it is a multifactorial condition, which develops during the diestrus as a consequence of ascending opportunistic bacterial infection [11,12,14]. A previous study developed by our research group demonstrated an association between PEH and pyometra in female dogs, whereas no significant association was demonstrated between pyometra and cystic endometrial hyperplasia [7]. This observation raised the hypothesis that tissue changes in PEH uterus can predispose the uterus to infection and inflammation.

Toll-like receptors (TLR) are transmembrane proteins that play a role during the initial steps of the innate immune response by recognizing pathogen associated molecular patters (PAMPs) [15,16]. TLRs 1–9 are expressed by canine endometrial cells at all phases of estrous cycle [17]. TLR2, which may form heterodimers with TLR1 or TLR6, is mainly responsible for recognizing molecules from fungi and gram-positive bacteria, whereas TLR4 is the mainly responsible for recognition of lipopolysaccharide (LPS) from gram-negative bacteria [15,16]. Therefore, studies investigating the canine uterine innate immune response, particularly in the context of the pathogenesis of pyometra, focused principally on TLR2 and TLR4 [17–19].

Other important mediators of innate immunity are interleukins and chemokines that act simultaneously to facilitate adhesion and migration of leukocytes driving these cells to the site of inflammation [20,21]. Studies investigating cytokines involved in canine pyometra demonstrated that mainly interleukin 6 (IL-6) and the chemokine (C-X-C motif) ligands such as CXCL8, CXCL5, and CXCL10 are involved [22–24]. IL-6 is a multifunctional interleukin involved in immune and inflammatory response by activating and directing the traffic of leukocytes, especially neutrophils. IL-6 also induces production of acute-phase proteins by hepatocytes, triggering systemic inflammatory response [21]. CXCL8 and CXCL5 are involved in neutrophil chemotaxis, while CXCL10 is a monocyte/macrophage chemoattractant [20, 24, 25].

Many pathological conditions that affect the canine female reproductive system are influenced or triggered by hormonal changes, including neoplasms such as mammary gland tumors, cystic endometrial hyperplasia, and uterine inflammation [10,11]. Female dogs are monoestrous and polytocous mammals, with spontaneous ovulation and a long period of anestrus that follows each estrous cycle. Additionally, female dogs have a prolonged diestrus with elevated progesterone levels regardless the establishment of pregnancy and the presence of embryos or fetuses within the uterus [26]. Under progesterone stimulation, the canine uterus is more prone to bacterial infections, since progesterone induces reduction of myometrial contraction, decreased blood flux to the uterus, and impairment of neutrophil diapedesis in the endometrium. Furthermore, the uterus under the effect of progesterone has lower levels of expression and transcription of TLR4, TLR2, and Interferon gamma, which are important players of the uterine immune response in dogs [17,27].

It has been recently demonstrated that there is no correlation between PEH and bacterial virulence patterns in bacteria isolated from cases of naturally occurring canine pyometra [28]. Our previous studies [7,29] support the hypothesis that PEH may be a predisposing factor for pyometra by changing the pattern of transcription and expression of inflammatory mediators in the uterus. Therefore, this study aimed to assess transcription levels and expression of TLRs (TLR1, TLR2, and TLR4), alpha estrogen receptor (ERS1), progesterone receptor (PR), and prolactin receptor (PRLR) by cultured uterine explants from dogs with PEH as well as the inflammatory response triggered by in vitro bacterial stimulation in cultured uterine tissues with PEH, which we demonstrated here that have a different pattern of receptors expression and proinflammatory response when compared to normal control uterine tissues.

## Materials and methods

### Ethics statement

This study was approved by the Institutional Animal Care and Use Committee at the Universidade Federal de Minas Gerais (UFMG), Belo Horizonte, Minas Gerais state, Brazil (protocol number CEUA-UFMG 173/2021). Additionally, an informed consent formulary was signed by all owners prior to tissue sampling.

### Animal sampling

Sample of the uterus, ovaries and vaginal swabs were obtained from healthy female dogs in diestrus, which had a history of at least one estrous, and were submitted to elective ovary-salpingo-hysterectomy (OSH) at the UFMG Veterinary Hospital. Animals with a recent history of antimicrobial or hormonal therapy were excluded from this study. Only dogs in diestrus were included in this study, being excluded dogs in proestrus, anestrus or estrus. A total of 23 animals were sampled, and 12 were included in the study. Exclusion criteria were any stage of the estrous cycle other than diestrus or any significant genital lesion.

**Fig 1:**
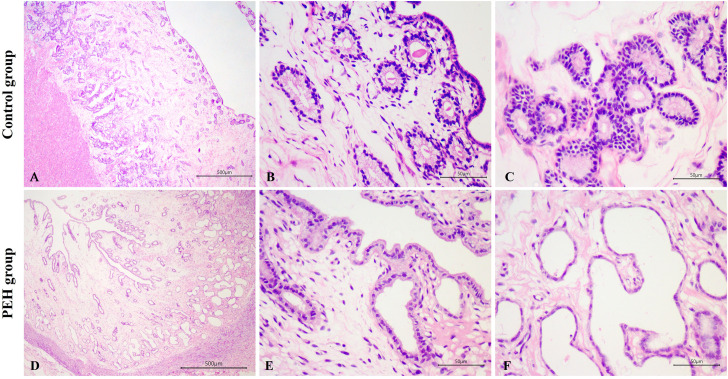
Uterine histology of dogs with pseudoplacentational endometrial hyperplasia (PEH) or unaffected controls. **(A)** Uterine morphologic features of the control group, without endometrial hyperplasia or glandular ectasia. HE, bar = 100 µm. **(B)** Cuboidal superficial endometrial epithelia. HE, bar = 50 µm. **(C)** Deep endometrial glands with cuboidal to columnar epithelium. HE, bar = 50 µm. **(D)** Uterine morphologic features of the PEH group, with endometrial hyperplasia and glandular dilatation. HE, bar = 500 µm. **(E)** Superficial endometrial epithelium and epithelium of superficial endometrial glands with columnar hyperplasic cells, with finely vacuolated cytoplasm, characterizing decidual reaction. HE, bar = 50 µm. **(F)** Deep endometrial glands with cuboidal to flattened epithelium, without decidual reaction. HE, bar = 50 µm.

Female dogs were divided into two groups according to their endometrial histological features, and dogs with endometrial or myometrial inflammation were excluded from this study. Groups were composed of (1) dogs with no uterine hyperplasic changes (control group; n = 6); (2) dogs with PEH (PEH group; n = 6) (Fig 1). PEH was histologically diagnosed as described [1,7,2]. Ages of dogs included in the PEH group ranged from 1.5 to 12 years (5.8 ± 1.6 years) whereas in the control group from 1.2 to 8 years (4.0 ± 1.23 years). All dogs from both PEH and control groups were in diestrus. Detailed information including breed, age, and experimental group of each individual dog included in this study is provided in the S1 Table.

### Identification of the phase of the estrous cycle

Immediately before OSH, vaginal swabs were obtained for cytological identification of estrous cycle phase at the time of de uterine sampling, according to Allison and colleagues [30]. Ovaries were grossly and microscopically evaluated to support the estrous cycle classification by vaginal cytology. Additionally, owners were questioned about the time of the last female dog heat, to indicate possible stage of estrous cycle.

### Histopathology

Uteri and ovaries were immediately sampled after OSH under sterile conditions. Fragments of both uterine horns were sampled, fixed in 10% buffered formalin for 24 to 48 hours, processed for paraffin embedding, sectioned (3 µm-thick sections), and stained with hematoxylin and eosin or further processed for immunohistochemistry.

### Immunohistochemical detection of Toll-like receptor 4 (TLR4) and hormonal receptors (PR, ESR1 and PRLR)

Immunohistochemistry was performed to evaluate localization and expression intensity of TLR4, ESR1, PR and PRLR receptors in uteri from control (n = 5) and PEH groups (n = 4), including explants in which transcription levels were measured. Samples that were not considered suitable for transcription analysis were excluded from this part of the study. Sections (3 µm-thick) of paraffin embedded tissues were mounted on charged slides, re-hydrated, submitted to antigen retrieval using a pressure cooker at 120^o^C for 10 min, with a high pH (pH = 9.0) solution (EnVision, USA) for PR and ESR1 antibodies and low pH (pH = 6.1) solution (EnVision, USA) for PRLR and TLR4 antibodies. Sections were then submitted to 30 min of peroxidase blockage, with commercial Peroxidase-Blocking Reagent (EnVision, USA), and 1 hour of non-specific protein binding blockage with 6% skimmed milk. Sections were incubated overnight in a dark chamber at 4ºC with the primary antibody. Specifications of antibodies and dilutions used are described in Table 1. Except for PRLR, sections were incubated 30 min in a dark chamber at room temperature with the secondary antibody with indirect peroxidase polymeric detection system (EnVision, USA), and reveled with HRP magenta substrate chromogen system (EnVision, Dako) and counter-stained with hematoxylin. For PRLR antibody, after primary antibody incubation, sections were incubated 30 min in a dark chamber at room temperature with the anti-goat IgG antibody. Specification and dilution of the anti-goat IgG antibody are described in Table 1. Sections were then incubated 30 min in a dark chamber at room temperature with the secondary antibody with indirect peroxidase polymeric detection system (EnVision, USA), revelation was performed with DAB chromogen (EnVision, USA) and counter-colored with hematoxylin. Negative controls had the primary antibody replaced by a commercial buffer washing solution (EnVision, USA).

**Table 1:** Antibodies and dilutions employed for immunohistochemistry in this study.

Target^*^	Antibody specifications	Immunohistochemistry Antibody dilution
TLR4	TLR4 antibody mouse monoclonal IgG1(clone 25), Santa Cruz Biotecnology, USA (catalog number: sc-293072)	1:1000
ESR1	Flex monoclonal rabbit anti-human estrogen receptor α (clone EP1) Ready-to-use, Dako, USA (catalog number: IR084)	Ready to use
PR	Rabbit polyclonal Anti-progesterone receptor antibody C-terminal, Abcam, USA (catalog number: ab191138)	1:400
PRLR	Anti-hProlactin R Affinity purified polyclonal Goat IgG, R&D Systems, USA (catalog number: AF1167)	1:50
Anti-goat IgG	Rabbit anti-goat IgG whole molecule FITC, Sigma-Aldrich, USA (catalog number: F7367)	1:500

* TLR4: Toll-like receptor 4; ESR1: Estrogen receptor alpha; PR: progesterone receptor; PRLR: prolactin receptor.

Immunolabeling was evaluated considering a score [31] with: immunolabeling intensity – (0) no stain, (1) weak, (2) moderate, and (3) strong; and frequency of positive immunolabelled cells – (0) no immunolabelled cells, (1) less them 20% of immunolabelled cells, (2) 21% to 50% of immunolabelled cells, (3) 51% to 80% of immunolabelled cells and (4) 81% to 100% of immunolabelled cells. Score analyses were performed separately in superficial endometrial epithelium, superficial endometrial glands, deep endometrial glands, stroma and myometrium. Immunoreactivity score was calculated by the sum of intensity and frequency scores at site (luminal epithelium, superficial glands, deep glands, stroma, and myometrium) and compared between groups. For the explants all 6 mm diameter tissue was evaluated and for uterine biopsy the complete uteri ring fragment, from at least three uterine fragments by case, were evaluated.

### Immunohistochemistry analyses of hormonal receptors (PR, ESR1 and PRLR) in archived uterine samples

To ensure that uterine explants were representative of in vivo tissues, immunohistochemistry was employed for assessing expression of ESR1, PR, and PRLR receptors in uterine biopsies from the UFMG veterinary pathology archive. Uteri (n = 21) were classified in two groups: PEH (n = 12) and normal uteri (n = 9), based on histologic evaluation. Immunohistochemistry protocols and the immunohistochemistry score classification were the same applied to uteri explants as described above. Archived uterine biopsies included 2–3 sections of each uterine horn, obtained from 2018 to 2021. Archived biopsies were evaluated at hematoxylin and eosin stain and only uterus without inflammation and appropriately fixed in formalin were included in the study.

### Stimulation and culture of uterine explants with heat killed *Escherichia coli*

The tissue culture protocol was adapted from Borges and colleagues [32]. Viability of cultured uterine explants has been previously tested and the uterine tissue remained viable up to 12 hours in culture. Explants were stimulated with the equivalent of 10^8^ colony forming units (CFU) of heat killed *Escherichia coli* and remained viable up to 12 hours in culture. Tissue viability was assessed histologically, considering maintenance of and integrity of luminal epithelium, superficial and deep endometrial glands, stroma, and myometrium (S1 Fig). Furthermore, reverse transcriptase quantitative polymerase chain reaction (RT-qPCR) quantification of constitutive genes was also evaluated as an indicator of tissue viability, and no decrease in transcription levels of glyceraldehyde-3-phosphate dehydrogenase (GAPDH) was detected from 6 to 12 hours in culture.

Twenty-four uterine explants, from each animal, were obtained under sterile conditions using a 6-mm diameter punch through all uterine layers, including endometrium, myometrium and perimetrium. Explants were washed once with Hank’s balanced salt solution (HBSS 1X Gibco, USA) supplemented with 5% of 200 UI/mL penicillin and 2 mg/mL streptomycin (Penicillin-Streptomycin Gibco, USA), and 2.5 µg/mL of amphotericin B (Gibco, USA) and twice with pure HBSS. After washing explants were placed in 24-well culture plaques with 2 mL, by well, of Roswell Park Memorial Institute (RPMI) 1,640 Medium (Gibco, USA) with 10% of sterile and filtered fetal bovine serum (Nova Biotecnologia, Brazil) and 1.1% of 100 mM sodium pyruvate (Gibco, USA), supplemented with 5% of 200 UI/mL penicillin and 2 mg/mL streptomycin (Penicillin-Streptomycin Gibco, Invitrogen) and 2.5 µg/mL of amphotericin B (Gibco, USA).

To evaluate the uterine inflammatory response to bacterial stimulation, explants of each animal were cultivated for 6 hours (n = 12 explants) or 12 hours (n = 12 explants), being half of them stimulated with equivalent 10^8^ CFU/mL of heat inactivated *E. coli* and the other half with sterile RPMI 1,640 medium. The strain sample of *E. coli* was gently donated by the anaerobic bacteriology laboratory from EV-UFMG [33]. It was isolated from a naturally infected female dog with pyometra and classified as *E. coli* phylogroup B2, which is the most frequent phylogroup isolated from canine pyometra [28]. Bacteria inactivation was performed by heating for 45 min at 75ºC. Inactivation was confirmed by seeding one aliquot of inactivated bacteria on Luria-Bertani (LB) agar, incubated at 37ºC and 5% of CO_2_ for 48 hours, evaluating the plates by each 12 hours.

After each time of incubation explants were half sampled and conditionate at 10% buffered formalin, for histopathological and immunohistochemistry, and half sampled and stored in a RNAlater solution (Invitrogen, USA) at -80^o^C for RNA extraction. All supernatant media were stored at -20^o^C for ELISA. Explants sampling and culture protocol are schematized in (S2 Fig).

### Transcription levels analyses of cytokines, toll-like (TLR) and hormonal receptors

The RNA extraction was performed with TRIzol reagent (Invitrogen, USA) and cleaned with DNase I RNase-free (Invitrogen, USA) following the manufacturer’s instructions. cDNA synthesis was performed using 1,000 ng of total RNA using the High-Capacity cDNA Reverse Transcription Kits (Applied Biosystems, USA), with random oligonucleotides and RNase inhibitor, following the manufacturer’s instructions.

Transcription levels of *CXCL8* and *IL-6* was assessed by RT-qPCR. Non-stimulated explants cultured for 6 hours were evaluated for *TLR1*, *TLR2*, *TLR4*, *ESR1*, *PR* and *PRLR* tissue transcription levels. GAPDH was defined as housekeeping gene. All qPCR reactions were performed using 96-well optical reaction plates (Applied Biosystems, USA) and the Applied Biosystems StepOnePlus Real Time PCR thermocycler, with 6.25 µL of Power SYBR Green PCR Master Mix (Applied Biosystems, USA), 0.5 µL of each forward (10 µM) and reverse (10 µM) oligonucleotide sequencies and 3.75 µL of commercial diethyl-pyrocarbonate-treated water (Invitrogen, USA), in a total reaction of 12.5 µL. Oligonucleotides sequencies used are described in Table 2. Oligonucleotides designed in this study were designed using the Primer3 Plus software (https://www.primer3plus.com/index.html), *in silico* tested with NCBI data (https://www.ncbi.nlm.nih.gov/), and biological tested using known positive control samples.

**Table 2:** primer sequences for each target gene and length of the amplicon generated in base pairs (bp).

Target gene^*^	Oligonucleotides sequencies	Amplicon length	Reference/ GenBank accession
*CXCL8*	F: 5′-CTCTGTGTGAAGCTGCAGTTCTGTC-3′ R: 5′-ATTTGGGGTGGAAAGGTGTGG-3′	90 bp	This study NM_001003200.1
*IL-6*	F: 5′- AACCTACATCTTCCCAAACTGG-3′ R: 5′- TGTAGCTGAAACTCCACAAGAC-3′	110 bp	Sasidharan and colleagues [34]
*TLR1*	F: 5′-TGCCATCCTACCGTGAACCTCA-3′ R: 5′-ACTGTGTGGCACTCAACCCCAGA-3′	124 bp	Turchetti and colleagues [35]
*TLR2*	F: 5′-TCCGACACAAGAATGCAAAG-3′ R: 5′-GGCAAAATCAGGGAAAATGA-3′	101 bp	Turchetti and colleagues [35]
*TLR4*	F: 5′-GCCATTGCTTCTCCAACTTC-3′ R: 5′-TGGTTTAGGCCCTGATATGC-3′	97 bp	Turchetti and colleagues [35]
*PR*	F: 5′-CGAGTCATTACCTCAGAA GAT TTG-3′ R: 5′-CTTCCATTGCCCTTTTAAAGA AGA-3′	113 bp	Graubner and colleagues [36]
*ESR1*	F: 5′- TGTAGAGGGCATCGTGGAGA −3′ R: 5′- CCTCGCCCTGGAGATTCATC- 3′	80 bp	This study NM_001286958.2
*PRLR*	F: 5′-GGATCTTTGTGGCCGTTCTTT-3′ R: 5′-AAGGATGCAGGTCACCATGCTAT-3′	92 bp	Graubner and colleagues [36]
*GAPDH*	F: 5′-AAGGCTGAGAACGGGAAACT-3′ R: 5′-TACTCAGCACCAGCATCACC-3′	101 bp	Turchetti and colleagues [35]

* TLR: Toll-like receptor; IL-6: interleukin 6; CXCL8: chemokine 8; ESR1: estrogen receptor alpha; PR: progesterone receptor; PRLR: prolactin receptor; GAPDH: glyceraldehyde-3-phosphate dehydrogenase.

Transcription data were evaluated by relative transcription levels by fold change analyses. Transcription levels of *TLR* and hormonal receptors from PEH group were normalized by both *GAPDH* and control group transcription levels. For pro-inflammatory analyses of *IL-6* and *CXCL8*, transcription levels from control and PEH groups were normalized by both *GAPDH* and transcription levels in non-stimulated explants, from each group, by each time point in culture.

### Concentration of CXCL8 from uterine explants supernatant by sandwich ELISA analyses

Detection and quantification of CXCL8 in supernatants of cultured uterine explants from control group (n = 6) and PEH group (n = 6) was performed with DuoSet ELISA Development system Canine IL-8/CXCL8 (R&D Systems, USA) kit following the manufacturer’s instructions. This ELISA kit has a detection limit of 4.31 pg/mL, and intra-assay and inter-assay coefficient of variations in the range of 5.2–6.0 and 4.7–5.9, respectively. Briefly, sandwich ELISA reactions were performed using 96-well plates. Plates were sensibilized with capture antibody (4 µg/mL), and a standard curve was included in all reactions, ranging from 1,000 pg/mL to 15.6 pg/mL. Supernatant protein was detected with detection antibody (0.25 µg/mL), reactions revealed with HRP conjugated streptavidin reagent, for 20 min, followed by the addition of substrate (0.1 M anhydrous citric acid, 0.2 M sodium phosphate, 0.05% o-phenylenediamine dihydrochloride (OPD) and 0.1% H_2_O_2_), for 5 min, and stopped with 2 N H_2_SO_4_. Resulting absorbance was recorded in an ELISA reader at 450 nm (MR-96A Microplate reader, Mindray, China) and converted to concentrations based on the standard curve. All assays were performed in duplicate and negative controls were included in all reactions.

### Statistical analyses

Transcription levels data were logarithmic normalized, and statistical analyses were performed by Student *t* test at GraphPad Prism 8.0.1, with significant differences when p < 0.05. Immunohistochemistry scores were compared by the non-parametric Mann-Whitney U test at GraphPad Prism 8.0.1, with significant differences considering p < 0.05. Immunoreactive score, intensities and frequencies of immunostaining were compared between groups. Means of optic density (OD) were converted to concentration (pg/mL), based on standard curve OD, data logarithmic normalized and compared between groups by Student t tes*t* at GraphPad Prism 8.0.1, with differences being considered significant when p < 0.05.

## Results

### Transcription levels of toll-like receptors 1, 2, and 4; and hormonal receptors

**Fig 2:**
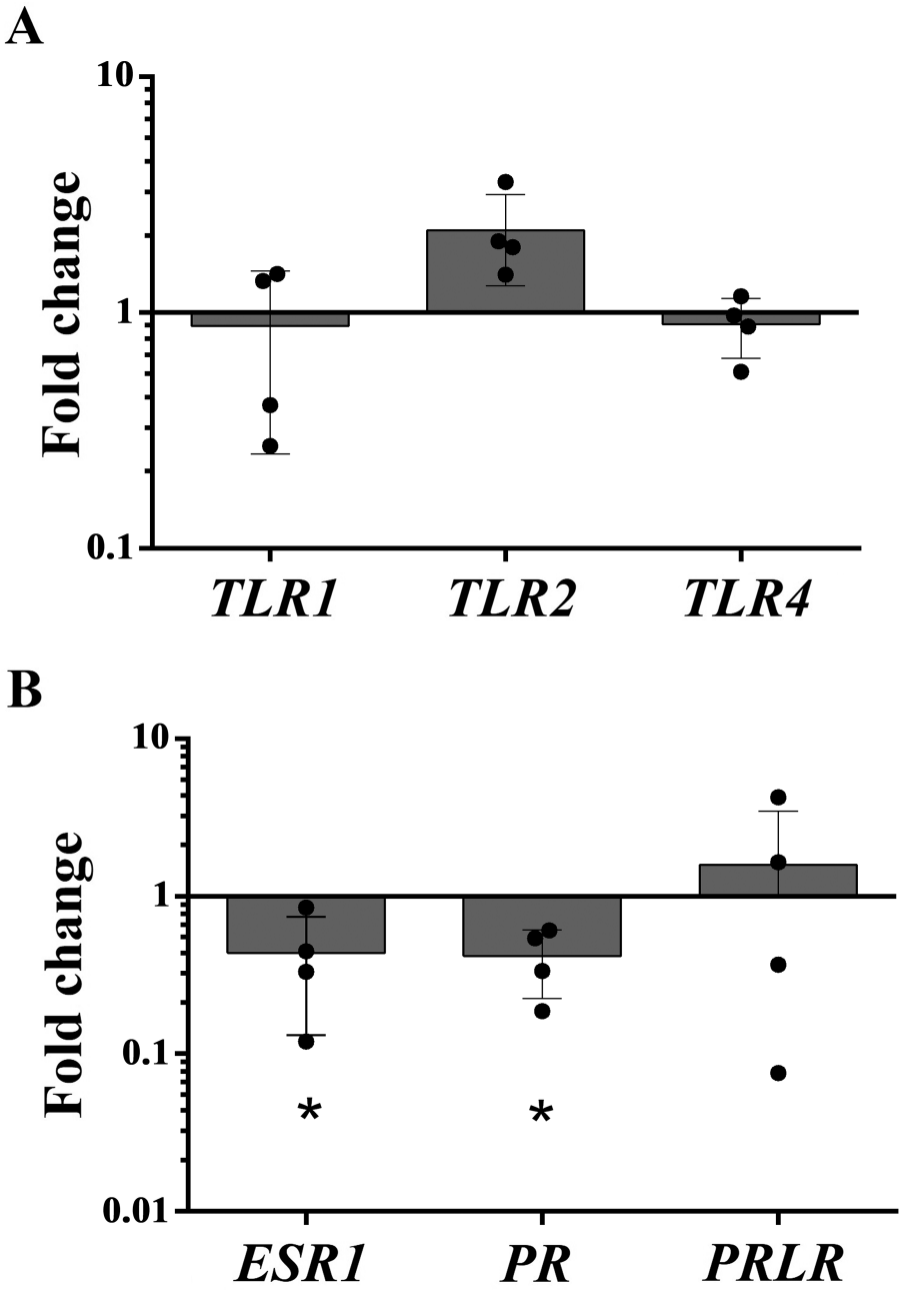
Transcription levels of Toll-like receptors (*TLR*) *1*, *TLR2* and *TLR4* and hormonal receptors: estrogen alpha receptor (*ESR1*), progesterone receptor (*PR*), and prolactin receptor (*PRLR*) in uterine explants from controls (n = 5) or pseudoplacentational endometrial hyperplasia (PEH) (n = 4) groups. **(A)** Fold change in transcription levels of *TLR1*, *TLR2*, and *TLR4* in uteri from the PEH group. **(B)** Fold change in transcription levels of *ESR1*, *PR*, and *PRLR* in uteri from the PEH group. Data are given as fold change of transcripts in the PEH group in relation to the control group. Means were compared by the Student *t* Test (* p < 0.05).

Non stimulated explants of control (n = 5) and PEH (n = 4) groups were used to evaluate the relative transcription levels of uterine receptors at 6 hours in culture. Transcription levels of *TLR1*, *TLR2*, and *TLR4* were not significantly different between PEH and controls (Fig 2A). Mean fold change for *ESR1*, *PR*, and *PRLR* in PEH group were, approximately, 0.44, 0.41, and 1.58, respectively. *ESR1* and *PR* transcription levels were significantly lower in PEH uterine explants compared to controls (Fig 2B).

### Expression of TLR4 and hormonal receptors in uterine explants

**Fig 3:**
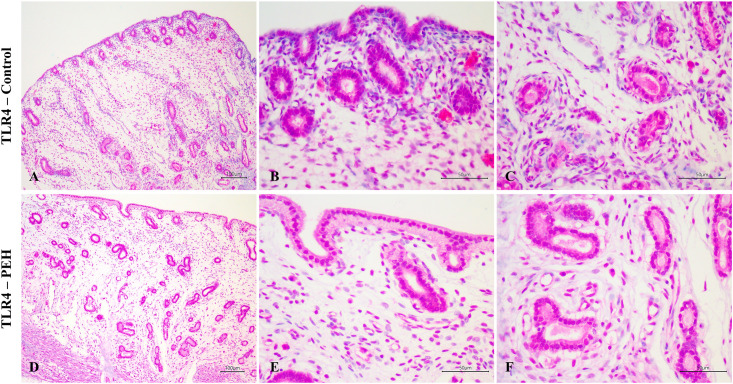
Expression of Toll-like receptor 4 (TLR4) as assessed by immunohistochemistry in canine uterine explants with either no changes (control group, n = 5) or pseudoplacentational endometrial hyperplasia (PEH group, n = 4). **(A-C)** TLR4 expression in uterus from control group with strong expression at all layers except at stromal cells. Magenta chromogen, bars = 100 µm (A) 50 µm **(B and C)**. **(D-F)** TLR4 expression in uteri from PEH group with strong expression in all layers except at stromal cells. Magenta chromogen, bars = 100 µm **(D)**, 50 µm **(E and F)**.

**Fig 4:**
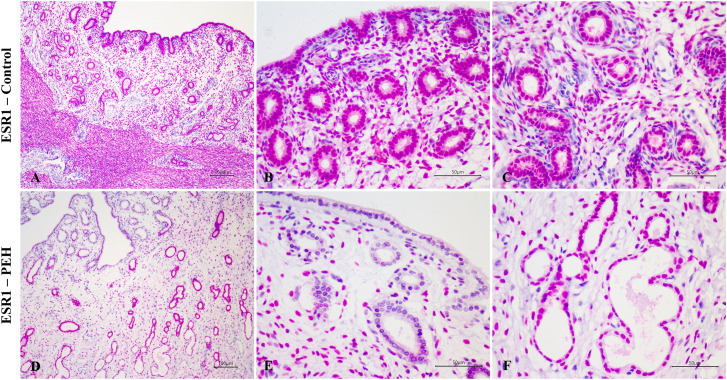
Expression of alpha estrogen receptor (ESR1) as assessed by immunohistochemistry in canine uterine explants with either no changes (control group, n = 5) or pseudoplacentational endometrial hyperplasia (PEH group, n = 4). **(A-C)** ESR1 expression in uteri from control group with strong expression in all layers. Magenta chromogen, bars = 100 µm **(A)**, 50 µm **(B and C)**. **(D-F)**. ESR1 expression in uteri from PEH group with weak and less frequent expression in luminal epithelium, superficial endometrial glands and in dilated deep glands. Magenta chromogen, bars = 500 µm **(D)**, 50 µm **(E and F)**.

**Fig 5:**
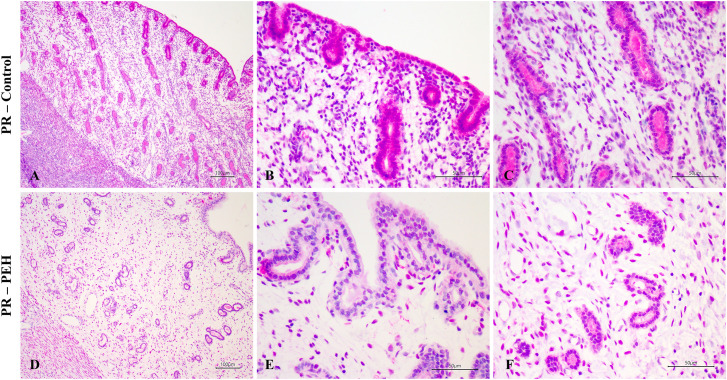
Expression of progesterone receptor (PR) as assessed by immunohistochemistry in canine uterine explants with either no changes (control group, n = 5) or pseudoplacentational endometrial hyperplasia (PEH group, n = 4). **(A – C)** PR expression in uteri from control group with strong expression in all layers. Magenta chromogen, bars = 100 µm **(A)**, 50 µm **(B and C)**. **(D – F)** PR expression in uteri from PEH group with weak and less frequent expression in luminal epithelium and superficial endometrial glands. Magenta chromogen, bars = 100 µm **(D)**, 50 µm **(E and F)**.

**Fig 6:**
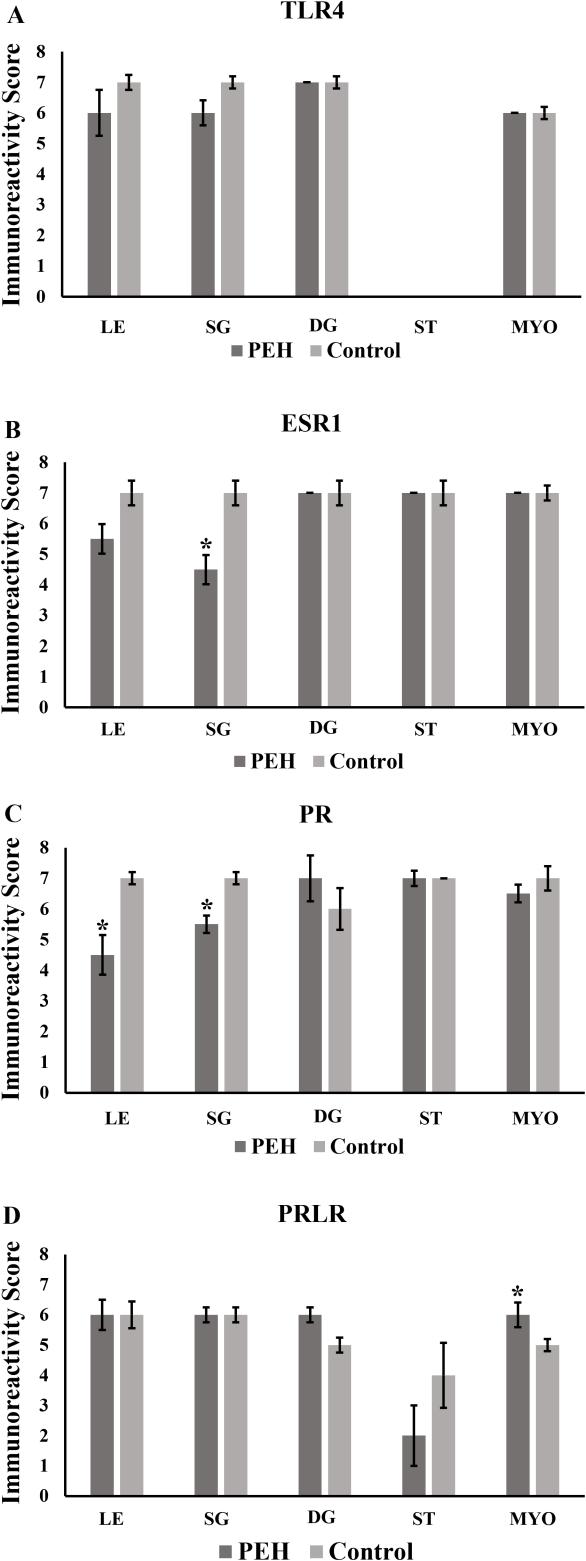
Expression of Toll-like receptor 4 (TLR4), estrogen receptor alfa (ESR1), progesterone receptor (PR) and prolactin receptor (PRLR) as assessed by immunohistochemistry in canine uterine explants with either no changes (control group, n = 5) or pseudoplacentational endometrial hyperplasia (PEH group, n = 4). Immunoreactivity score was calculated by the sum of intensity (I) and frequency (F) scores of immunostaining. **(A)** Immunoreactivity score for TLR4 expression. **(B)** Immunoreactivity score for ESR1 expression. **(C)** Immunoreactivity score for PR expression. **(D)** Immunoreactivity score for PRLR expression. Medians were compared in each localization by Mann-Whitney U test (* p > 0.05). LE: luminal epithelium; SG: superficial endometrial glands; DG: deep endometrial glands; ST: stroma and MYO: myometrium.

Expression of TLR4, assessed by immunohistochemistry, both in control and PEH groups, was demonstrated in many uterine tissue components including the luminal epithelium, superficial glands, deep glands, and myometrium. No expression was observed in endometrial stromal cells in both groups. No differences of immunoreactivity score, intensity or frequency were observed between groups (Figs 3 and 6). Expression of both ESR1 and PR was observed in all uterine layers (Figs 4 and 5), whereas expression of PR was observed only in the luminal epithelium and superficial glands (Figs 5 and 6). ESR1 expression observed in superficial glands was lower in explants with PEH (Figs 4 and 6). Detailed score results are presented in S2 Table.

**Fig 7:**
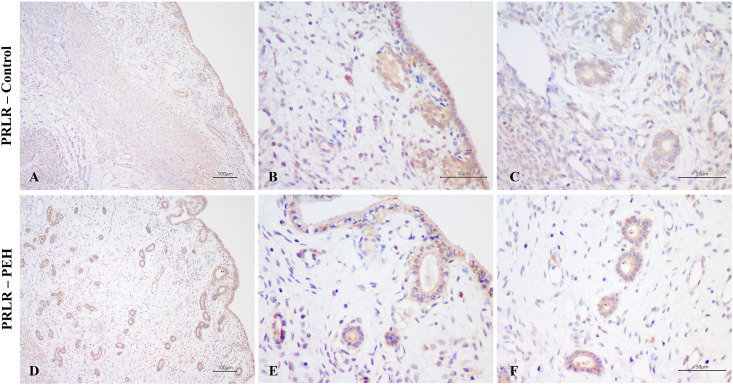
Expression of prolactin receptor (PRLR) as assessed by immunohistochemistry in canine uterine explants with either no changes (control group, n = 5) or pseudoplacentational endometrial hyperplasia (PEH group, n = 4). **(A – C)** PRLR expression in uteri from control group with moderate to weak expression in all layers. DAB, bars = 100 µm **(A)**, 50 µm **(B and C)**. **(D – F)** PRLR expression in uteri from PEH group with moderate to weak expression in all layers. DAB, bars = 100 µm **(D)**, 50 µm **(E and F)**.

Considering intensity and frequency of immunostaining, expressions of both PR and ESR1 were significantly less intense in the luminal epithelium and superficial glands of explants with PEH (S3 Fig), particularly in luminal and glandular epithelial cells with decidual reaction. Furthermore, expression of PR was less frequent in the luminal epithelium of explants with PEH (S3 Fig). PRLR expression was markedly variable in both groups, with higher intensity of expression in PEH myometrium (Fig 7 and S3 Fig).

### Localization and intensity of expression of hormonal receptors in archived uterine biopsies

**Fig 8:**
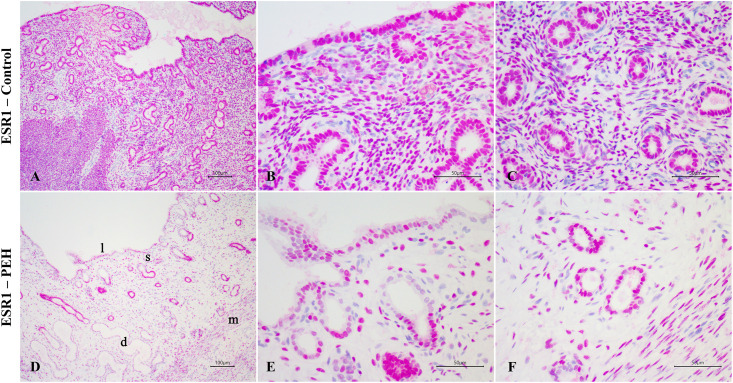
Expression of alpha estrogen receptor (ESR1) as assessed by immunohistochemistry in uteri from the archive samples from dogs with pseudoplacentational endometrial hyperplasia (PEH; n = 12) and control uteri (control; n = 9). **(A – C)** ESR1 expression in control uterus with strong expression in all layers. Magenta chromogen, bars = 100 µm **(A)**, 50 µm **(B and C)**. **(D-F)** ESR1 expression in PEH uterus with lower expression in luminal epithelium **(l)**, superficial endometrial glands **(s)**, dilated deep glands **(d)**, and myometrium **(m)**. Magenta chromogen, bars = 100 µm **(D)**, 50 µm **(E and F)**.

**Fig 9:**
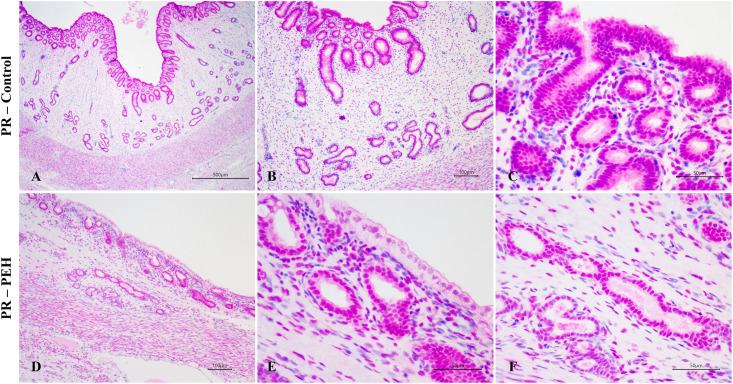
Expression of progesterone receptor (PR) as assessed by immunohistochemistry in uteri from the archive samples from dogs with pseudoplacentational endometrial hyperplasia (PEH; n = 12) and control uteri (control; n = 9). **(A – C)** PR expression in control uterus with strong expression in all layers. Magenta chromogen, bars = 100 µm **(A and B)**, 50 µm **(C)**. **(D – F)** PR expression in PEH uterus with lower expression intensity in luminal epithelium. Magenta chromogen, bars = 100 µm **(D)**, 50 µm **(E and F)**.

**Fig 10:**
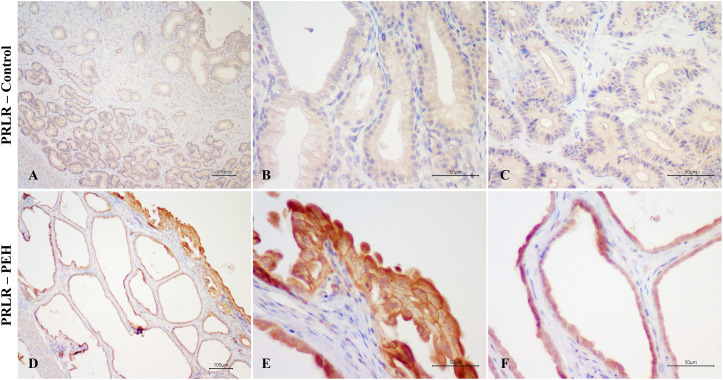
Expression of prolactin receptor (PRLR) as assessed by immunohistochemistry in uteri from the archive samples from dogs with pseudoplacentational endometrial hyperplasia (PEH; n = 10) and control uteri (control; n = 5). **(A – C)** PRLR expression in control uterus with low intensity in all layers. DAB, bars = 100 µm **(A)**, 50 µm **(B and C)**. **(D – F)** PRLR expression. In PEH uterus with strong intensity in luminal epithelium and superficial endometrial glands. DAB, bars = 100 µm **(D)**, 50 µm **(E and F)**.

**Fig 11:**
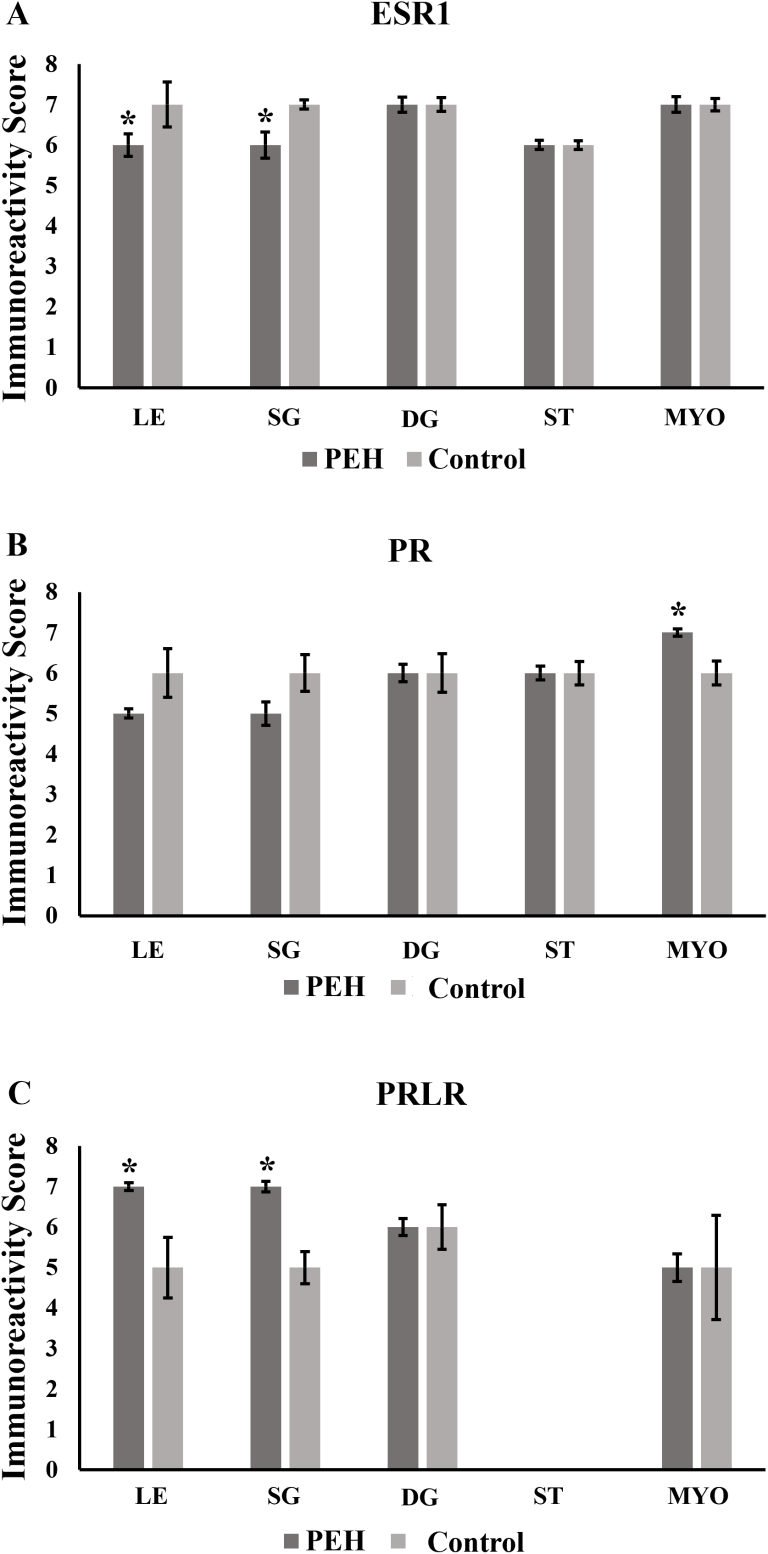
Expression of estrogen receptor alfa (ESR1), progesterone receptor (PR) and prolactin receptor (PRLR) as assessed by immunohistochemistry in dogs with pseudoplacentational endometrial hyperplasia (PEH, n = 12 for ESR1 and PR; n = 10 for PRLR) and control uteri (control, n = 9 for ESR1 and PR; n = 5 for PRLR) in uteri from the archive samples. Immunoreactivity score (SI) was calculated by the sum of intensity (I) and frequency (F) of immunostaining. **(A)** Immunoreactivity score for ESR1 expression. **(B)** Immunoreactivity score for PR expression. **(C)** Immunoreactivity score for PRLR expression. Medians were compared in each localization by Mann-Whitney U test, (* p > 0.05). LE: luminal epithelium; SG: superficial endometrial glands; DG: deep endometrial glands; ST: stroma and MYO: myometrium.

Expression of PR and ESR1 were assessed by immunohistochemistry in uterine biopsies from both PEH (n = 12) and control uteri (n = 9), and PRLR in PEH (n = 10) and control (n = 5) uteri. Detailed results are presented in S3 Table. Expression of ESR1 was less intense in the luminal epithelium and superficial glands of uteri with PEH (Figs 8–11 and S4 Fig). Expression of PR had no differences in immunoreactivity scores in the luminal epithelium and superficial glands (Fig 11). However, the intensity of immunostaining in luminal epithelium was lower in PEH (S4 Fig). Interestingly, both immunoreactivity score and frequency of PR expression were lower in the myometrium from dogs with PEH (Fig 11 and S4 Fig). Tissue expression of PRLR by immunochemistry demonstrated higher expression of PRLR at luminal epithelium and superficial glands in PEH uterus (Fig 10), considering both immunoreactivity score and intensity (Fig 11 and S4 Fig).

### Transcription levels of *CXCL8* and *IL-6* in uterine explants after stimulus by heat killed *Escherichia coli*

**Fig 12:**
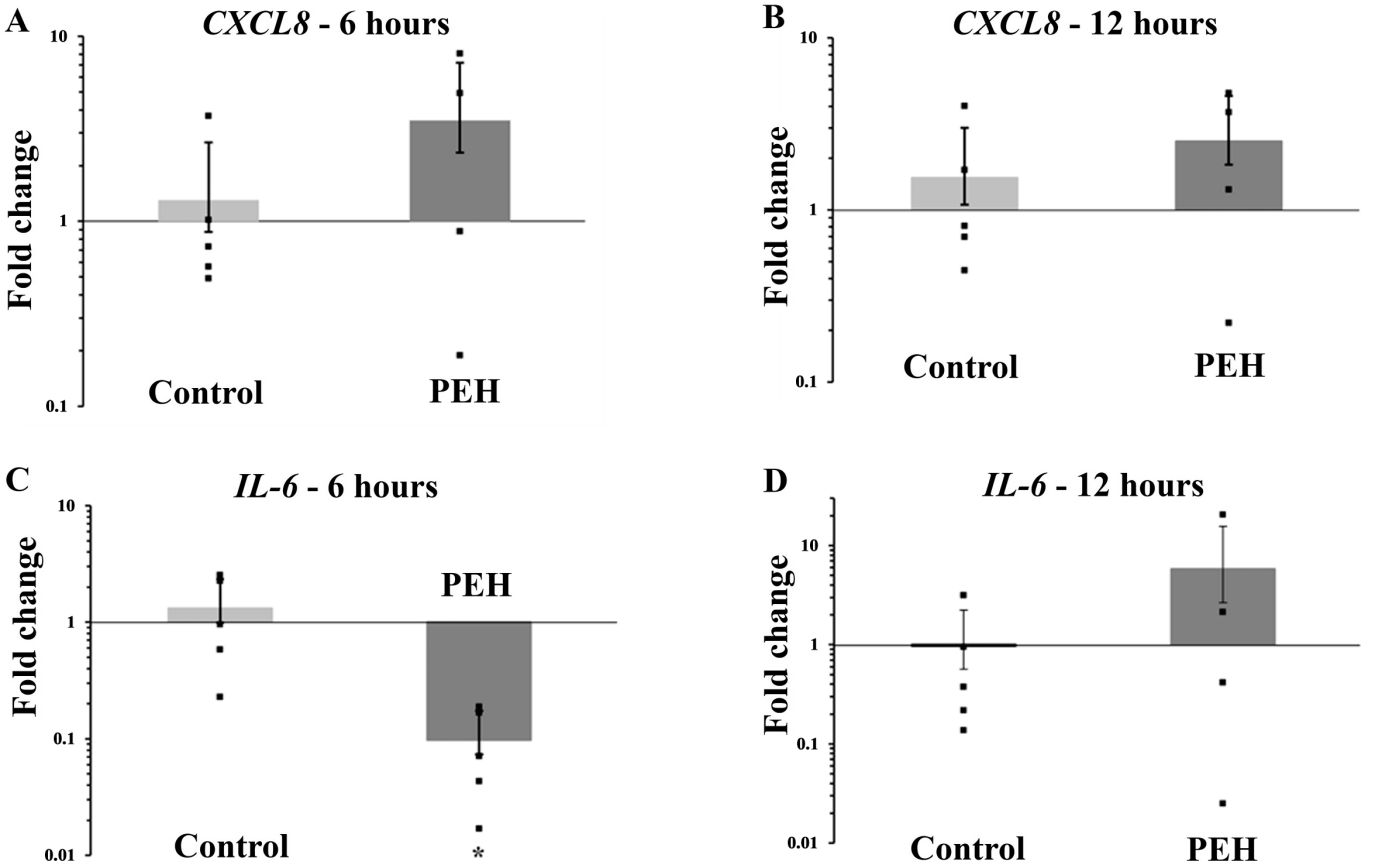
Transcription levels of *CXCL8* and *IL-6* in uterine explants from control (n = 5) and pseudoplacentational endometrial hyperplasia (PEH) (n =4) groups, stimulated in vitro with the equivalent of 10^8^ CFU of heat killed *Escherichia coli* for 6 and 12 hours post stimulation. **(A)** Fold changes of *CXCL8* transcription levels in control and PEH groups, at 6 hours post stimulation. **(B)** Fold changes of *CXCL8* transcription levels in control and PEH groups, at 12 hours post stimulation. **(C)** Fold changes of *IL-6* transcription levels in control and PEH groups, at 6 hours post stimulation. **(D)** Fold change of *IL-6* transcription levels in control and PEH groups, at 12 hours post stimulation. Fold changes were compared by Student *t* Test (* p < 0.05).

Uterine explants from control group (n = 5) and PEH group (n = 4) stimulated with the equivalent of 10^8^ CFU of heat killed *E. coli*, for 6 and 12 hours, were evaluated for assessing transcription levels of *CXCL8* and *IL-6*. There were no significant differences in transcription levels of *CXCL8* when control tissues were compared to PEH explants at 6 and 12 hours post stimulation (Fig 12A and 12B ). In contrast, at 6 hours post stimulation transcription levels of *IL-6* were significantly lower in PEH in comparison to controls (Fig 12C), indicating downregulation of *Il6* in PEH explants after stimulation with inactivated *E. coli*. At 12 hours post stimulation, no statistical difference was observed for *IL-6* transcription levels between control and PEH groups. However, the pattern of transcription levels from 6 to 12 hours in PEH group was clearly different, with an inversion from downregulation to upregulation of *IL-6* transcription levels in PEH explants, with 64.0 fold change when stimulated PEH explants were compared between 6 and 12 hours post stimulation (Fig 12C and 12D ). The control group had minimal or no *IL-6* induction either at 6 or 12 hours post stimulation.

### Production of CXCL8 by cultured uterine explants after stimulus by heat killed *Escherichia coli*

**Fig 13:**
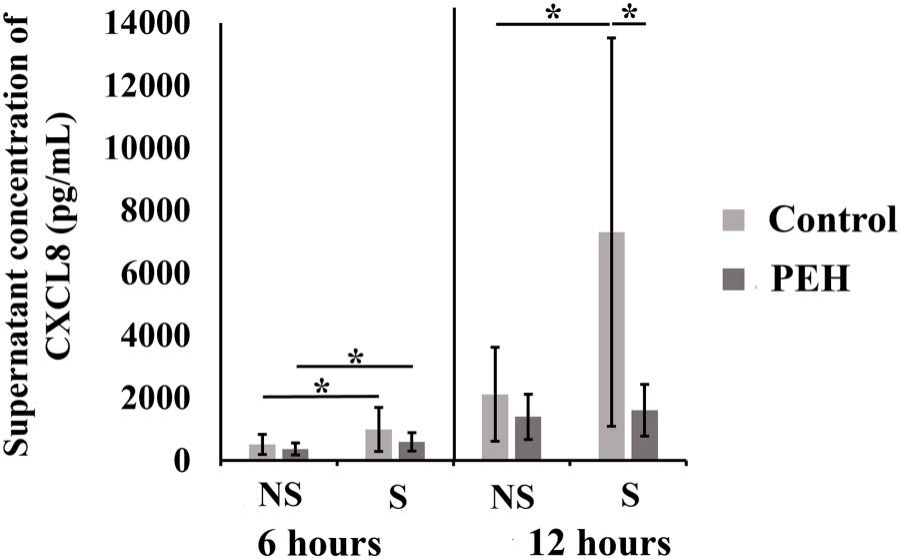
CXCL8 secretion (pg/mL) from uterine explants from control (n = 6) or pseudoplacentational endometrial hyperplasia (PEH) groups (n = 6). Uterine explants were stimulated in vitro with the equivalent of 10^8^ CFU of heat killed *Escherichia coli* for 6 and 12 hours post stimulation. Concentrations were normalized and the means of groups data were compered at each time point by Student *t* test (* p > 0.05). NS: non stimulated explants; S: stimulated explants. Control: control group. PEH: pseudoplacentational endometrial hyperplasia group.

Supernatants from cultured uterine explants from control group (n = 6) and PEH group (n = 6) stimulated with the equivalent of 10^8^ CFU of heat killed *E. coli*, for 6 and 12 hours, were evaluated for measuring CXCL8. At 6 hours post stimulation, explants from both control and PEH groups had CXCL8 production in response to stimulation, with no differences between groups (Fig 13). However, concentrations of CXCL8 at 12 hours post stimulation were significantly diminished in the PEH group (Fig 13). In contrast, control explants maintained CXCL8 levels up to 12 hours post stimulation. Therefore, production of CXCL8 at 12 hours post stimulation was significantly higher in control uterine explants compared to explants with PEH (Fig 13).

## Discussion

This study demonstrated for the first time that uterine tissues with PEH have an altered pattern of expression and transcription of genes associated with hormonal regulation of the uterus and innate immunity, which is an important first step for a better understanding of a possible cause and effect relationship between PEH and pyometra in dogs. Uterus with PEH had both lower expression and lower transcription levels of ESR1 and PR, mostly characterized by the reduction of the expression intensity predominantly at decidualized cells from luminal epithelium and superficial endometrial glands. Similar pattern of ESR1 and PR expression was described in uterus from pregnant dogs at both non-placentation and placentation sites, with none or weak immunostaining of both receptors [37]. Lower expressions of ESR1 and PR suggest that uterine tissues with PEH are less responsive to these hormones. Importantly, the pattern of hormonal receptors expression corroborates the fact that PEH and CEH are different conditions not just based on morphology [7], but also functionally since in CEH expression of ESR1 is increased [38]. Therefore, in contrast to the pathogenesis of CEH in which high serum estrogen stimulates endometrial hyperplasia [9,39], probably estrogen is not involved in the development of PEH.

Our results support the notion that uterine tissues with PEH may be less affected by high serum progesterone concentrations during diestrus. However, the frequency of PR expression in myometrial cells was higher in uterus from the biopsies archive which indicates that myometrial contraction may be reduced in cases of PEH, impairing a protective response against bacterial infection [11]. In this context of predisposition to infection, studies verified that female dog uterus with pyometra has low transcription levels of *ESR1*, suggesting that the low response to estrogen can be related to bacterial infection susceptibility [38,40]. Low transcription levels and expression of *ESR1* in the PEH uterus may also be a predisposing factor to pyometra. However, the pattern of hormonal receptors expression in PEH uterus from the present study, considering ESR1, PR and PRLR, is completely different from these other reports.

Archived uterine biopsies with PEH had stronger expression of PRLR at both luminal epithelium and superficial glands when compared to unaffected uterine tissues. Interestingly, cells with stronger expression were the cells with decidual reaction. There is no previous study evaluating PRLR expression in uterus from non-pregnant dog. However, transcription of PRLR were observed in uterus and placenta from pregnant dogs during pre-implantation, post-implantation until mid-gestation, with significantly increasing levels through those gestational stages [41]. Additionally, strong expression of PRLR was detected in the epithelium from endometrium and endometrial glands at interplacental polar zones, and at endometrial epithelium, gland and placenta at placentation sites [41] which suggest expression patterns in PEH mimics the pregnant uterus. However, this hypothesis needs to be further investigated.

Transcription levels of *CXCL8* after stimulation with inactivated *E. coli* demonstrated no differences between PEH and control groups, either at 6 or 12 hours. Furthermore, supernatant concentrations of CXCL8 corroborated that at 6 hours both groups had similar CXCL8 proinflammatory response to the stimulation. Interestingly, at 12 hours PEH explants do not respond with CXCL8 production once no difference was observed between non stimulated and stimulated PEH explants. Thus, for some reason, PEH tissue ceased the CXCL8 production earlier then control tissue having a shorter CXCL8 proinflammatory response. Transcription levels of *IL-6* had a different pattern from *CXCL8* in the PEH group explants, with significant downregulation of *IL-6* at 6 hours post stimulation followed increased transcription levels at 12 hours post stimulation, with upregulation of 64.0 times more than at 6 hours. The transcription level pattern in PEH group suggests that *IL-6* is probably an important cytokine involved in PEH uterine response to bacterial stimuli and can be the target for further understanding PEH inflammation in pyometra cases. Interestingly, the pattern of the inflammatory response observed at PEH uteri resemble the innate immune response observed in uterus from pregnant dogs at peri-implantation [42]. In uteri from pregnant dogs, in addition to other immune response factors, right before and right after implantation there is increasing of IL6 and IL-1β availability, while availability of CXCL8 progressively and markedly decreases from pre to post implantation [43].

This study expands the knowledge on canine PEH, including hormonal receptors transcription and expression and proinflammatory response to bacterial stimulus. A better understanding of mechanisms leading to PEH and its consequences, particularly its role on the pathogenesis of canine pyometra, may eventually provide better therapeutic approaches to this highly prevalent and potentially lethal condition.

## Conclusions

Uterus with PEH had different pattern of hormonal receptors expression, with lower transcription levels of *ESR1* and *PR*. Expression of ESR1 and PR were lower at superficial endometrial epithelium and superficial endometrial glands in PEH uterus, which correspond to the location of epithelial cells with a decidual reaction, while PRLR expression levels at those sites were higher. Proinflammatory response to bacterial stimulus was characterized by a shorter CXCL8 production in PEH uterus and different pattern of *IL-6* transcription in comparison to unaffected control uterine tissues.

## Supporting information

S1 Fig Representative histology of dog uterine explants. Cultivation viability analyses of dog uterine explants, cultured with Roswell Park Memorial Institute (RPMI) 1,640 Medium (Gibco, USA) with 10% of sterile and filtered fetal bovine serum (Nova Biotecnologia, Brazil) and 1.1% of 100 mM sodium pyruvate (Gibco, USA), supplemented with 5% of 200 UI/mL penicillin and 2 mg/mL streptomycin (Penicillin-Streptomycin Gibco, USA) and 2.5 µg/mL of amphotericin B (Gibco, USA), during 6 hours (C and D), 12 hours (E and F), 24 hours (G and H) and 48 hours (I and J). (A) Histology of non-cultured uterine tissue with preserved luminal epithelium, superficial endometrial glands, (B) and deep endometrial glands. (C) Histology of explant cultured by 6 hours with preserved luminal epithelium, superficial endometrial glands, (D) and deep endometrial glands. (E) Histology of explant cultured by 12 hours with preserved luminal epithelium, superficial endometrial glands, (F) and deep endometrial glands. (G) Histology of explant cultured by 24 hours with preserved luminal epithelium, important loss of the structure of superficial endometrial glands, (H) and deep endometrial glands (red arrows). (I) Histology of explant cultured by 48 hours with preserved luminal epithelium, completely loss of the structure of superficial endometrial glands, (J) and deep endometrial glands. Hematoxylin and eosin, 200X (A, B, C, D, E, G, H and I) and 400X (F and J).
(TIF)

S2 Fig Schema of explants sampling and culture protocol. (1) Mesometrium was removed. (2) Horns were opened, and explants obtained with a 6-mm diameter punch through all uterine layers. (3) Explants were washed once with Hank’s balanced salt solution (HBSS) supplemented with 5% of 200 UI/mL penicillin and 2 mg/mL streptomycin and twice with pure HBSS. (4) Explants were individually placed in 24-well culture plaques with 2 mL, by well, of Roswell Park Memorial Institute (RPMI) 1,640 medium with 10% of sterile and filtered fetal bovine serum and 1.1% of 100 mM sodium pyruvate, supplemented with 5% of 200 UI/mL penicillin and 2 mg/mL streptomycin and 2.5 µg/mL of amphotericin B. (5) Half of the explants were stimulated with equivalent 10^8^ CFU/mL of heat inactivated *Escherichia coli* and the other half with sterile RPMI 1,640 medium. Explants were kept during 6 or 12 hours in culture. (6) After each time of incubation explants were half conditionate at 10% buffered formalin, for histopathological and immunohistochemistry, and half sampled and stored in RNAlater solution.
(TIF)

S3 Fig Intensity (I) and frequency (F) expression of Toll-like receptor 4 (TLR4), estrogen receptor alfa (ESR1), progesterone receptor (PR) and prolactin receptor (PRLR) as assessed by immunohistochemistry in canine uteri with either no changes (control group, n = 5) or pseudoplacentational endometrial hyperplasia (PEH group, n = 4). (A) Immunoreactivity score for TLR4 expression. (B) Intensity and frequency for TLR4 expression. (C) Immunoreactivity score for ESR1 expression. (D) Intensity and frequency for ESR1 expression. (E) Immunoreactivity score for PR expression. (F) Intensity and frequency for PR expression. (G) Immunoreactivity score for PRLR expression. (H) Intensity and frequency for PRLR expression. Medians were compared in each localization by Mann-Whitney U test (* p > 0.05). LE: luminal epithelium; SG: superficial endometrial glands; DG: deep endometrial glands; ST: stroma and MYO: myometrium.
(TIF)

S4 Fig Intensity (I) and frequency (F) expression of estrogen receptor alfa (ESR1), progesterone receptor (PR) and prolactin receptor (PRLR) as assessed by immunohistochemistry in dogs with pseudoplacentational endometrial hyperplasia (PEH, n = 12 for ESR1 and PR; n = 10 for PRLR) and control uteri (control, n = 9 for ESR1 and PR; n = 5 for PRLR) in uteri from the archive samples. (A) Immunoreactivity score for ESR1 expression. (B) Intensity and frequency for ESR1 expression. (C) Immunoreactivity score for PR expression. (D) Intensity and frequency for PR expression. (E) Immunoreactivity score for PRLR expression. (F) Intensity and frequency for PRLR expression. Medians were compared in each localization by Mann-Whitney U test, (* p > 0.05). LE: luminal epithelium; SG: superficial endometrial glands; DG: deep endometrial glands; ST: stroma and MYO: myometrium.
(TIF)

S1 Table Breed, age, and group of each individual dog included in this study. (DOCX)

S2 Table Immunohistochemistry score from To*l*l-like receptor 4 (TLR4), estrogen receptor alpha (ESR1), progesterone receptor (PR) and prolactin receptor (PRLR) evaluated in control and PEH experimental groups. Medians and standard error (described in parenthesis) from intensity (I), frequency (F) and immunoreactivity score (IS) from endometrial luminal epithelium (LE), superficial endometrial glands (SG), deep endometrial glands (DG), stroma (ST) and myometrium (MYO).
(DOCX)

S3 Table Immunohistochemistry score from estrogen receptor alpha (ESR1), progesterone receptor (PR) and prolactin receptor (PRLR) evaluated in control and PEH dogs uterus from biopsies archive. Medians and standard error (described in parenthesis) from intensity (I), frequency (F) and immunoreactivity score (IS) from endometrial luminal epithelium (LE), superficial endometrial glands (SG), deep endometrial glands (DG), stroma (ST) and myometrium (MYO).
(DOCX)
