# Supplementary figures and images for: Expression of hormonal receptors and Toll-like receptors in cultured canine uterine explants with pseudoplacentational endometrial hyperplasia and bacterial-elicited endometrial inflammation
Source: PLoS One. 2025 Sep 5;20(9):e0331209. doi: 10.1371/journal.pone.0331209 (PMC12412960; doi:10.1371/journal.pone.0331209)

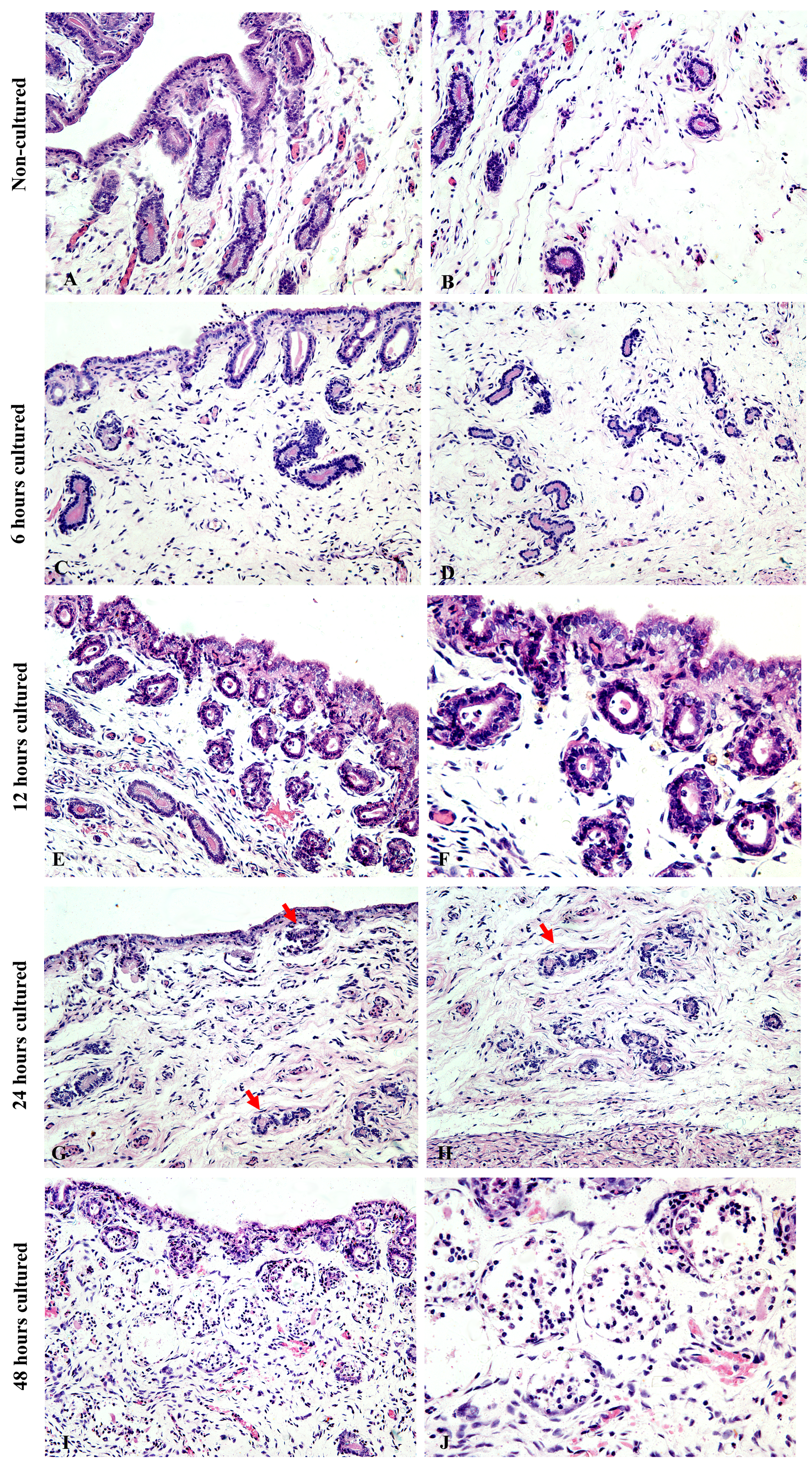

Supplement: S1 Fig — Cultivation viability analyses of dog uterine explants, cultured with Roswell Park Memorial Institute (RPMI) 1,640 Medium (Gibco, USA) with 10% of sterile and filtered fetal bovine serum (Nova Biotecnologia, Brazil) and 1.1% of 100 mM sodium pyruvate (Gibco, USA), supplemented with 5% of 200 UI/mL penicillin and 2 mg/mL streptomycin (Penicillin-Streptomycin Gibco, USA) and 2.5 µg/mL of amphotericin B (Gibco, USA), during 6 hours (C and D), 12 hours (E and F), 24 hours (G and H) and 48 hours (I and J). (A) Histology of non-cultured uterine tissue with preserved luminal epithelium, superficial endometrial glands, (B) and deep endometrial glands. (C) Histology of explant cultured by 6 hours with preserved luminal epithelium, superficial endometrial glands, (D) and deep endometrial glands. (E) Histology of explant cultured by 12 hours with preserved luminal epithelium, superficial endometrial glands, (F) and deep endometrial glands. (G) Histology of explant cultured by 24 hours with preserved luminal epithelium, important loss of the structure of superficial endometrial glands, (H) and deep endometrial glands (red arrows). (I) Histology of explant cultured by 48 hours with preserved luminal epithelium, completely loss of the structure of superficial endometrial glands, (J) and deep endometrial glands. Hematoxylin and eosin, 200X (A, B, C, D, E, G, H and I) and 400X (F and J). (TIF) [file pone.0331209.s001.tif]

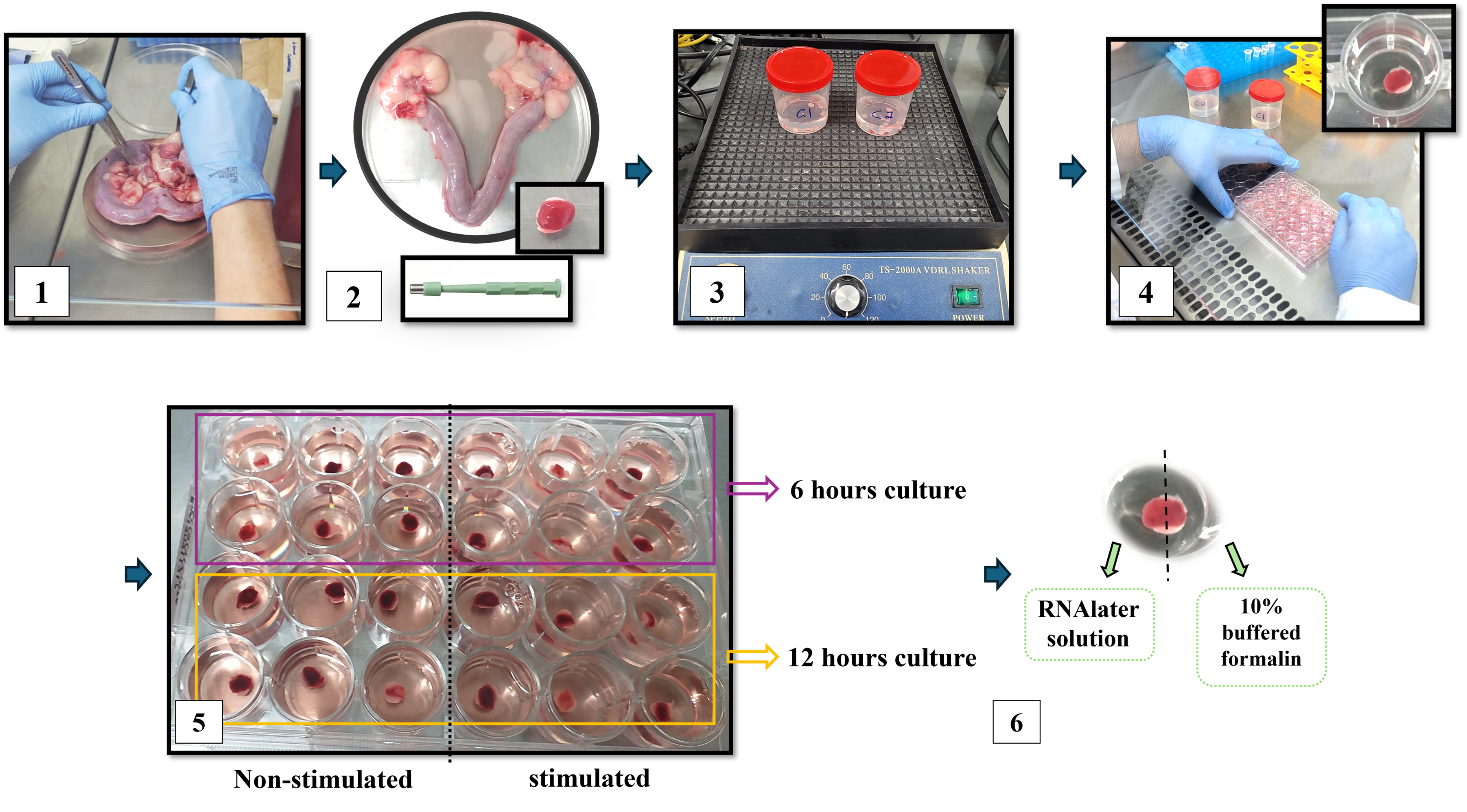

Supplement: S2 Fig — (1) Mesometrium was removed. (2) Horns were opened, and explants obtained with a 6-mm diameter punch through all uterine layers. (3) Explants were washed once with Hank’s balanced salt solution (HBSS) supplemented with 5% of 200 UI/mL penicillin and 2 mg/mL streptomycin and twice with pure HBSS. (4) Explants were individually placed in 24-well culture plaques with 2 mL, by well, of Roswell Park Memorial Institute (RPMI) 1,640 medium with 10% of sterile and filtered fetal bovine serum and 1.1% of 100 mM sodium pyruvate, supplemented with 5% of 200 UI/mL penicillin and 2 mg/mL streptomycin and 2.5 µg/mL of amphotericin B. (5) Half of the explants were stimulated with equivalent 108 CFU/mL of heat inactivated Escherichia coli and the other half with sterile RPMI 1,640 medium. Explants were kept during 6 or 12 hours in culture. (6) After each time of incubation explants were half conditionate at 10% buffered formalin, for histopathological and immunohistochemistry, and half sampled and stored in RNAlater solution. (TIF) [file pone.0331209.s002.tif]

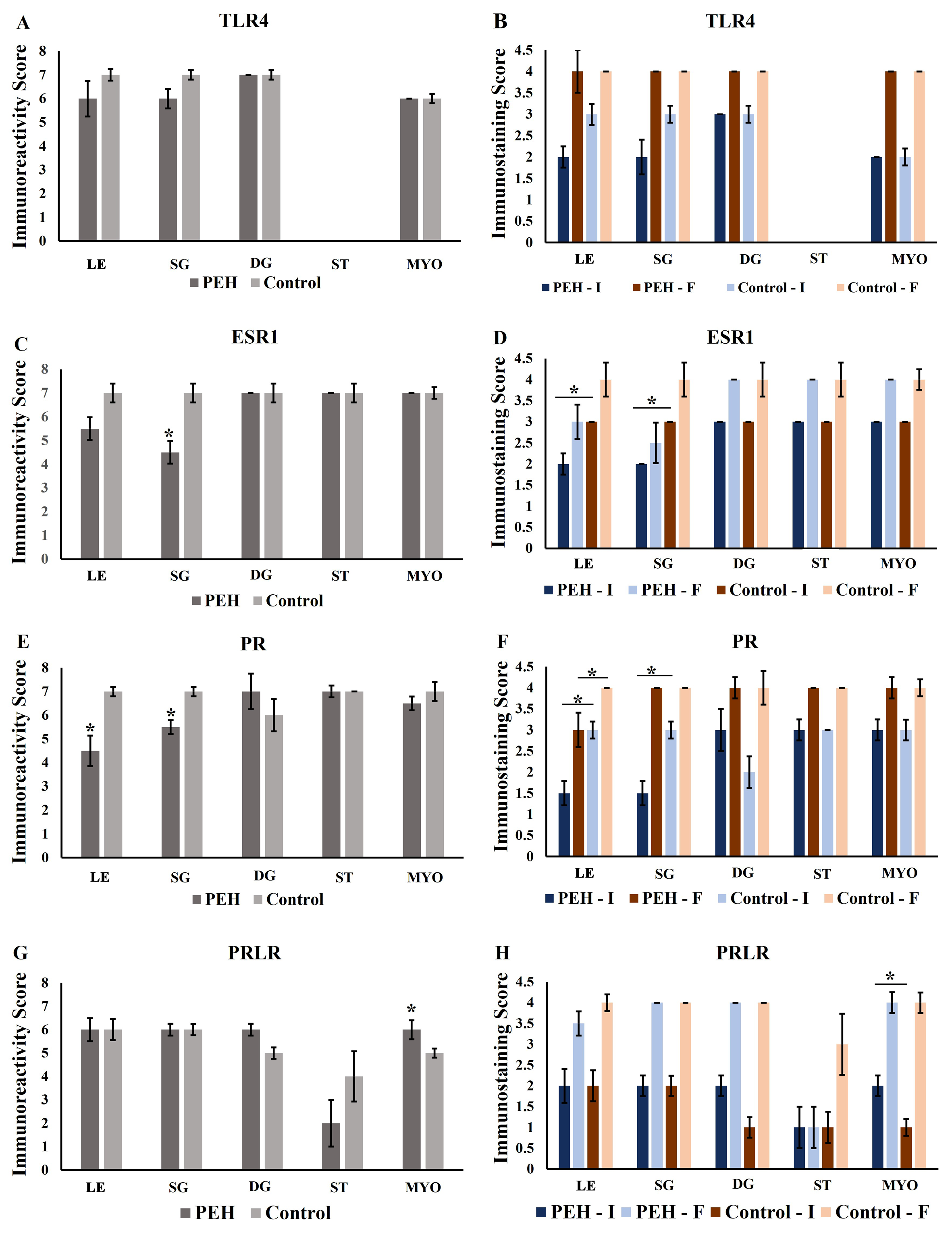

Supplement: S3 Fig — (A) Immunoreactivity score for TLR4 expression. (B) Intensity and frequency for TLR4 expression. (C) Immunoreactivity score for ESR1 expression. (D) Intensity and frequency for ESR1 expression. (E) Immunoreactivity score for PR expression. (F) Intensity and frequency for PR expression. (G) Immunoreactivity score for PRLR expression. (H) Intensity and frequency for PRLR expression. Medians were compared in each localization by Mann-Whitney U test (* p > 0.05). LE: luminal epithelium; SG: superficial endometrial glands; DG: deep endometrial glands; ST: stroma and MYO: myometrium. (TIF) [file pone.0331209.s003.tif]

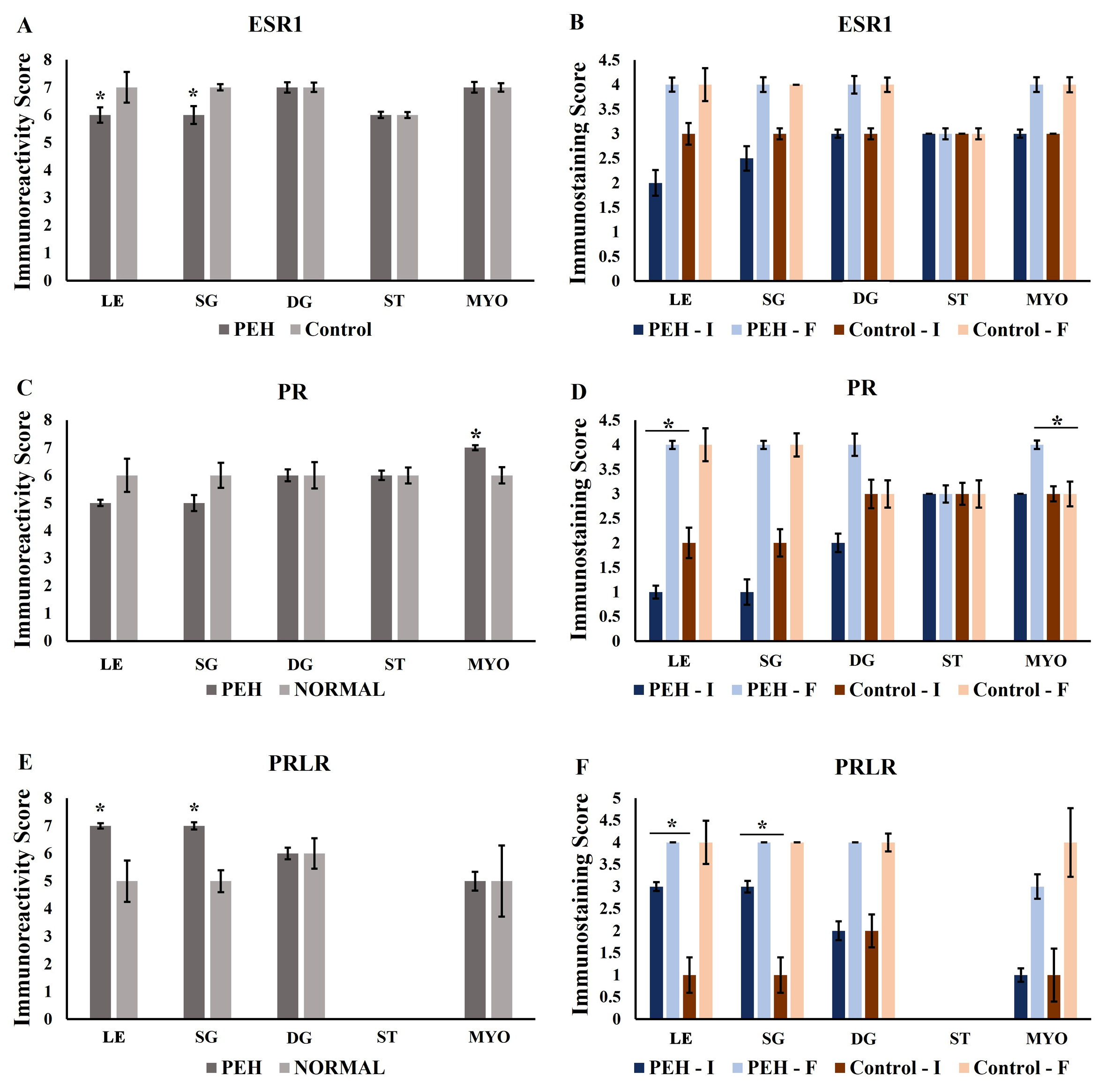

Supplement: S4 Fig — (A) Immunoreactivity score for ESR1 expression. (B) Intensity and frequency for ESR1 expression. (C) Immunoreactivity score for PR expression. (D) Intensity and frequency for PR expression. (E) Immunoreactivity score for PRLR expression. (F) Intensity and frequency for PRLR expression. Medians were compared in each localization by Mann-Whitney U test, (* p > 0.05). LE: luminal epithelium; SG: superficial endometrial glands; DG: deep endometrial glands; ST: stroma and MYO: myometrium. (TIF) [file pone.0331209.s004.tif]
