# Supplementary material for: Expression of hormonal receptors and Toll-like receptors in cultured canine uterine explants with pseudoplacentational endometrial hyperplasia and bacterial-elicited endometrial inflammation
Source: PLoS One. 2025 Sep 5;20(9):e0331209. doi: 10.1371/journal.pone.0331209 (PMC12412960; doi:10.1371/journal.pone.0331209)
Supplement: S1 Table — (DOCX) [file pone.0331209.s005.doc]

**Supplementary Table S1.** Breed, age, and group of each individual dog included in this study.

| **Dog ID** | **Breed** | **Age (years)** | **Group** |
| --- | --- | --- | --- |
| A02 | Mixed breed | 1.4 | Control |
| A05 | Pinscher | 4.0 | PEH |
| A07 | Gigant Schnauzer | 5.9 | Control |
| A08 | Shih Tzu | 1.3 | Control |
| A09 | Mixed breed | 9.0 | PEH |
| A11 | Poodle | 1.5 | PEH |
| A12 | Dalmatian | 5.0 | PEH |
| A13 | Mixed breed | 8.0 | Control |
| A15 | Mixed breed | 1.2 | Control |
| A16 | Mixed breed | 12.0 | PEH |
| A17 | Mixed breed | 6.0 | Control |
| A23 | Border Collie | 3.3 | PEH |
