# Supplementary material for: Expression of hormonal receptors and Toll-like receptors in cultured canine uterine explants with pseudoplacentational endometrial hyperplasia and bacterial-elicited endometrial inflammation
Source: PLoS One. 2025 Sep 5;20(9):e0331209. doi: 10.1371/journal.pone.0331209 (PMC12412960; doi:10.1371/journal.pone.0331209)
Supplement: S2 Table — Medians and standard error (described in parenthesis) from intensity (I), frequency (F) and immunoreactivity score (IS) from endometrial luminal epithelium (LE), superficial endometrial glands (SG), deep endometrial glands (DG), stroma (ST) and myometrium (MYO). (DOCX) [file pone.0331209.s006.doc]

**Supplementary Table S2.** Immunohistochemistry score from To*l*l-like receptor 4 (TLR4), estrogen receptor alpha (ESR1), progesterone receptor (PR) and prolactin receptor (PRLR) evaluated in control and PEH experimental groups. Medians and standard error (described in parenthesis) from intensity (I), frequency (F) and immunoreactivity score (IS) from endometrial luminal epithelium (LE), superficial endometrial glands (SG), deep endometrial glands (DG), stroma (ST) and myometrium (MYO).

|  | **Analyses localization** | | | | | | | | | | | | | | |
| --- | --- | --- | --- | --- | --- | --- | --- | --- | --- | --- | --- | --- | --- | --- | --- |
| **Experimental groups** | **Parameter evaluated** | | | | | | | | | | | | | | |
|  | LE | | | SG | | | DG | | | ST | | | MYO | | |
| Antibody target  Sample group | I | F | IS | I | F | IS | I | F | IS | I | F | IS | I | F | IS |
| **TLR4** |  |  |  |  |  |  |  |  |  |  |  |  |  |  |  |
| PEH group | 2 (0.25) | 4 (0.50) | 6 (0.75) | 2 (0.41) | 4  (0) | 6 (0.41) | 3  (0) | 4  (0) | 7  (0) | 0  (0) | 0  (0) | 0  (0) | 2  (0) | 4  (0) | 6  (0) |
| Control group | 3 (0.24) | 4  (0) | 7 (0.24) | 3 (0.20) | 4  (0) | 7 (0.20) | 3 (0.20) | 4  (0) | 7 (0.20) | 0  (0) | 0  (0) | 0  (0) | 2 (0.20) | 4  (0) | 6 (0.20) |
| **ESR1** |  |  |  |  |  |  |  |  |  |  |  |  |  |  |  |
| PEH group | 2^a^ (0.25) | 3 (0.41) | 5.5 (0.48) | 2^a^  (0) | 2.5 (0.48) | 4.5^a^ (0.48) | 3  (0) | 4  (0) | 7  (0) | 3  (0) | 4  (0) | 7  (0) | 3  (0) | 4  (0) | 7  (0) |
| Control group | 3^b^  (0) | 4 (0.40) | 7 (0.40) | 3^b^  (0) | 4 (0.40) | 7^b^ (0.40) | 3  (0) | 4 (0.40) | 7 (0.40) | 3  (0) | 4 (0.40) | 7 (0.40) | 3  (0) | 4 (0.24) | 7 (0.24) |
| **PR** |  |  |  |  |  |  |  |  |  |  |  |  |  |  |  |
| PEH group | 1.5^a^ (0.29) | 3^a^ (0.41) | 4.5 (0.65) | 1.5^a^ (0.29) | 4  (0) | 5.5^a^ (0.29) | 3 (0.50) | 4 (0.25) | 7 (0.75) | 3 (0.25) | 4  (0) | 7 (0.25) | 3 (0.25) | 4 (0.25) | 6.5 (0.29) |
| Control group | 3^b^ (0.20) | 4^b^  (0) | 7 (0.20) | 3^b^ (0.20) | 4  (0) | 7^b^ (0.20) | 2 (0.37) | 4 (0.40) | 6 (0.68) | 3  (0) | 4  (0) | 7  (0) | 3 (0.24) | 4 (0.2) | 7 (0.4) |
| **PRLR** |  |  |  |  |  |  |  |  |  |  |  |  |  |  |  |
| PEH group | 2 (0.41) | 3.5 (0.29) | 6 (0.5) | 2 (0.25) | 4  (0) | 6 (0.25) | 2 (0.25) | 4  (0) | 6 (0.25) | 1 (0.50) | 1 (050) | 2  (1.0) | 2^a^ (0.25) | 4 (0.25) | 6^a^ (0.41) |
| Control group | 2 (0.37) | 4 (0.2) | 6 (0.45) | 2 (0.24) | 4  (0) | 6 (0.24) | 1 (0.24) | 4  (0) | 5 (0.24) | 1 (1.08) | 3 (0.37) | 4 (0.73) | 1^b^ (0.20) | 4 (0.24) | 5^b^ (0.2) |

^a, b^ Different letters in the between PEH and control groups for the same antibody indicate statistically significant differences (p < 0.05).
