# Supplementary material for: Expression of hormonal receptors and Toll-like receptors in cultured canine uterine explants with pseudoplacentational endometrial hyperplasia and bacterial-elicited endometrial inflammation
Source: PLoS One. 2025 Sep 5;20(9):e0331209. doi: 10.1371/journal.pone.0331209 (PMC12412960; doi:10.1371/journal.pone.0331209)
Supplement: S3 Table — Medians and standard error (described in parenthesis) from intensity (I), frequency (F) and immunoreactivity score (IS) from endometrial luminal epithelium (LE), superficial endometrial glands (SG), deep endometrial glands (DG), stroma (ST) and myometrium (MYO). (DOCX) [file pone.0331209.s007.doc]

**Supplementary Table** **S3.** Immunohistochemistry score from estrogen receptor alpha (ESR1), progesterone receptor (PR) and prolactin receptor (PRLR) evaluated in control and PEH dogs uterus from biopsies archives. Medians and standard error (described in parenthesis) from intensity (I), frequency (F) and immunoreactivity score (IS) from endometrial luminal epithelium (LE), superficial endometrial glands (SG), deep endometrial glands (DG), stroma (ST) and myometrium (MYO).

|  |  | **Analyses localization** | | | | | | | | | | | | | |
| --- | --- | --- | --- | --- | --- | --- | --- | --- | --- | --- | --- | --- | --- | --- | --- |
| **Uterus from biopsie archives** |  | **Parameter evaluated** | | | | | | | | | | | | | |
|  | LE | | | SG | | | DG | | | ST | | | MYO | | |
| Antibody target/ groups | I | F | IS | I | F | IS | I | F | IS | I | F | IS | I | F | IS |
| **ESR1** |  |  |  |  |  |  |  |  |  |  |  |  |  |  |  |
| PEH uterus | 2 (0.26) | 4 (0.14) | 6^a^ (0.28) | 2.5 (0.25) | 4 (0.15) | 6^a^ (0.32) | 3 (0.08) | 4 (0.18) | 7 (0.19) | 3  (0) | 3 (0.11) | 6 (0.11) | 3 (0.08) | 4 (0.15) | 7 (0.19) |
| Control uterus | 3 (0.22) | 4 (0.33) | 7^b^ (0.56) | 3 (0.11) | 4  (0) | 7^b^ (0.11) | 3 (0.11) | 4 (0.15) | 7 (0.17) | 3  (0) | 3 (0.11) | 6 (0.11) | 3  (0) | 4 (0.15) | 7 (0.15) |
| **PR** |  |  |  |  |  |  |  |  |  |  |  |  |  |  |  |
| PEH uterus | 1^a^ (0.13) | 4 (0.08) | 5 (0.11) | 1 (0.26) | 4 (0.08) | 5 (0.29) | 2 (0.19) | 4 (0.22) | 6 (0.21) | 3  (0) | 3 (0.17) | 6 (0.17) | 3  (0) | 4^a^ (0.09) | 7^a^ (0.09) |
| Control uterus | 2^b^ (0.31) | 4 (0.33) | 6 (0.60) | 2 (0.28) | 4 (0.24) | 6 (0.45) | 3 (0.29) | 3 (0.28) | 6 (0.47) | 3 (0.22) | 3 (0.28) | 6 (0.29) | 3 (0.15) | 3^b^ (0.25) | 6^b^ (0.30) |
| **PRLR** |  |  |  |  |  |  |  |  |  |  |  |  |  |  |  |
| PEH uterus | 3^a^ (0.10) | 4  (0) | 7^a^ (0.10) | 3^a^ (0.13) | 4  (0) | 7^a^ (0.13) | 2 (0.21) | 4  (0) | 6 (0.21) | 0  (0) | 0  (0) | 0  (0) | 1 (0.15) | 3 (0.28) | 5 (0.34) |
| Control uterus | 1^b^ (0.40) | 4 (0.49) | 5^b^ (0.70) | 1^b^ (0.40) | 4  (0) | 5^b^ (0.40) | 2 (0.37) | 4 (0.20) | 6 (0.50) | 0  (0) | 0  (0) | 0  (0) | 1 (0.60) | 4 (0.77) | 5 (1.30) |

^a, b^ Different letters in the between PEH and control groups for the same antibody indicate statistically significant differences (p < 0.05).
